# Supplementary material for: Lactic Acid Bacteria and Yeast Inocula Modulate the Volatile Profile of Spanish-Style Green Table Olive Fermentations
Source: Foods. 2019 Jul 24;8(8):280. doi: 10.3390/foods8080280 (PMC6723112; doi:10.3390/foods8080280)
Supplement: Supplementary file 1 [file foods-08-00280-s001.pdf]

Supplementary material to: *Lactic acid bacteria and yeast inocula modulate the volatile profile of Spanish-style green table olive fermentations*

**Table S1**. Volatile composition determined by GC-MS analysis in the brines of the different treatments assayed at the end of fermentation. T1 stand for treatment inoculated with LPG1, T2 inoculated with Lp13, T3 inoculated with Lpl15, T4 inoculated with Y12, and T5 inoculated with Y12+LPG1+Lp13+Lpl15.

| **Volatile compounds** | **LRI[a]** | **ID[b]** | **Relative peak area ± sd[c]** | | | | | |
|---|---|---|---|---|---|---|---|---|
| | | | **T1** | **T2** | **T3** | **T4** | **T5** | **T6 (Spontaneous)** |
| ***Acids*** | | | | | | | | |
| Acetic acid | 1444 | A | 0.10 ± 0.07[a] | 0.043 ± 0.004[a] | 0.03 ± 0.3[a] | 0.021 ± 0.002[a] | 0.08 ± 0.06[a] | 0.04 ± 0.03[a] |
| 2-Methylbutanoic acid | 1673 | B[1] | 0.092 ± 0.018[a] | 0.108 ± 0.010[a] | 0.06 ± 0.08[a] | 0.086 ± 0.008[a] | 0.13 ± 0.04[a] | 0.121 ± 0.018[a] |
| 3-Methylbutanoic acid | 1673 | A | 0.046 ± 0.008[a,b] | 0.038 ± 0.005[a,b] | 0.02 ± 0.03[a] | 0.034 ± 0.003[a,b] | 0.068 ± 0.017[b] | 0.037 ± 0.006[a,b] |
| Hexanoic acid | 1860 | A | 0.028 ± 0.020[a] | 0.026 ± 0.004[a] | 0.04 ± 0.6[a] | 0.011 ± 0.004[a] | 0.036 ± 0.024[a] | 0.037 ± 0.005[a] |
| 2-Ethylhexanoic acid | 1962 | B[2] | 0.0173 ± 0.0022[a] | 0.0097 ± 0.0015[a] | 0.010 ± 0.014[a] | 0.009 ± 0.011[a] | 0.012 ± 0.013[a] | 0.0130 ± 0.005[a] |
| Heptanoic acid | 1969 | A | 0.009 ± 0.010[a] | 0.0082 ± 0.0018[a] | 0.0 12 ± 0.017[a] | n.d.[a] | 0.09 ± 0.010[a] | 0.0104 ± 0.0015[a] |
| Octanoic acid | 2082 | A | 0.025 ± 0.017[a] | 0.019 ± 0.003[a] | n.d.[a] | 0.009 ± 0.003[a] | 0.025 ± 0.015[a] | 0.026 ± 0.005[a] |
| Decanoic acid | 2300 | A | 0.063 ± 0.017[a] | 0.026 ± 0.018[a] | 0.038 ± 0.011[a] | 0.028 ± 0.023[a] | 0.03 ± 0.03[a] | 0.04 ± 0.04[a] |
| *Total of acids* | | | 0.382[a] | 0.277[a] | 0.218[a] | 0.198[a] | 0.382[a] | 0.329[a] |
| ***Alcohols*** | | | | | | | | |
| Methanol | 886[a] | A | 0. 11 ± 0.04[a] | 0.175 ± 0.021[b] | 0.353 ± 0.009[c] | 0.126 ± 0.019[a,b] | 0.18 ± 0.04[b] | 0.169 ± 0.020[a,b] |
| Ethanol | 892[a] | A | 2.0 ± 0.5[a,c] | 1.89 ± 0.20[a] | 2.62 ± 0.05[a,c] | 3.9 ± 0.5[b] | 2.9 ± 0.5[b,c] | 2.3 ± 0.5[a,c] |
| 2-Butanol | 980[a] | B[3] | 0.058 ± 0.002[a] | 0.034 ± 0.003[b] | 0.041 ± 0.009[b,c] | 0.049 ± 0.006[a,c] | 0.060 ± 0.007[a] | 0.038 ± 0.004[b,c] |
| 2-Methyl-1-propanol | 1087 | A | 0.013 ± 0.011[a,b] | n.d.[a] | 0.0223 ± 0.0017[b] | 0.0137 ± 0.0012[a,b] | n.d.[a] | 0.021 ± 0.007[b] |
| 3-Pentanol | 1106 | A | 0.0178  ± 0.0014[a] | 0.017 ± 0.005[a] | 0.0207 ± 0.0004[a] | 0.022 ± 0.005[a] | 0.024 ± 0.003[a] | 0.022 ± 0.005[a] |
| 2-Pentanol | 1123 | A | 0.0108 ± 0.0022[a] | 0.01164 ± 0.00021[a] | 0.015 ± 0.003[a] | 0.0116 ± 0.0009[a] | 0.011 ± 0.003[a] | 0.01477 ± 0.00019[a] |
| 1-Butanol | 1147 | A | 0.0087 ± 0.0010[a] | 0.0071 ± 0.0005[a] | 0.0081 ± 0.0004[a] | 0.0209 ± 0.0012[b] | 0.0130  ± 0.0021[c] | n.d.[d] |
| 2-Methyl-1-butanol | 1209 | A | 0.23 ± 0.10[a,b] | 0.1208 ± 0.0022[a] | 0.293 ± 0.011[b] | 0.67 ± 0.07[c] | 0.282 ± 0.024[b] | 0.29 ± 0.07[b] |
| 3-Methyl-1-butanol | 1212 | A | 0.29 ± 0.18[a,b] | 0.082 ± 0.005[a] | 0.42 ± 0.06[b] | 0.88 ± 0.07[c] | 0.37 ± 0.04[b] | 0.37 ± 0.11[b] |
| 2-Hexanol | 1224 | A | 0.0091 ± 0.0011[a] | 0.0091 ± 0.0005[a] | 0.0094 ± 0.0008[a] | 0.088 ± 0.0007[a] | 0.0092 ± 0.0004[a] | 0.0097 ± 0.0005[a] |
| 5-Methyl-3-hexanol | 1237 | C | 0.0092 ± 0.0007[a] | 0.0085 ± 0.0003[a] | 0.005 ± 0.007[a] | 0.0099 ±0.0008[a] | 0.0091 ± 0.0003[a] | 0.0090 ± 0.0003[a] |
| 3-Methyl-3-buten-1-ol | 1247 | B[1] | 0.0204 ± 0.0017[a] | 0.0077 ± 0.0008[b] | 0.009 ± 0.003[b] | 0.0190 ± 0.0021[a] | 0.0199 ± 0.0019[a] | 0.0106 ± 0.0006[b] |
| 1-Pentanol | 1252 | A | 0.0111 ± 0.0006[a,d] | 0.0094 ± 0.0003[b] | 0.0100 ± 0.0010[a,b] | 0.0136 ± 0.0007[c] | 0.0121 ± 0.0009[c,d] | 0.0103 ± 0.0008[a,b] |
| *cis*-2-Penten-1-ol | 1319 | A | 0.0172 ± 0.0018[a] | n.d.[b] | n.d.[b] | 0.0169 ± 0.0014[a] | 0.017 ± 0.003[a] | n.d.[b] |
| 2-Methyl-2-buten-1-ol | 1320 | A | 0.0299 ± 0.0024[a] | 0.0189 ± 0.0005[b] | 0.021 ± 0.005[b,c] | 0.030 ± 0.004[a] | 0.028 ± 0.003[a,c] | 0.021 ± 0.004[b,c] |
| 1-Hexanol | 1351 | A | 0.180 ± 0.023[a,c,d] | 0.124 ± 0.009[b] | 0.134 ± 0.021[a,b] | 0.200 ± 0.009[c] | 0.19 ± 0.03[c,d] | 0.146 ± 0.006[a,b,d] |
| *cis*-3-Hexen-1-ol | 1379 | A | 0.57 ± 0.05[a] | 0.249 ± 0.009[b] | 0.29 ± 0.07[b] | 0.529 ± 0.025[a] | 0.58 ± 0.10[a] | 0.36 ± 0.03[b] |
| 5-Hexen-1-ol | 1405 | C | 0.0095 ± 0.0004[a] | 0.0095 ± 0.0009[a] | 0.0099 ± 0.0009[a] | 0.0111 ± 0.0003[a] | 0.0100 ± 0.0009[a] | 0.0100 ± 0.0013[a] |

| | | | | | | | | |
|---|---|---|---|---|---|---|---|---|
| 2-Methyl-3-hexanol | 1421 | C | 0.0063 ± 0.0009[a] | n.d.[b] | n.d.[b] | 0.0091 ± 0.0007[a] | 0.004 ± 0.005[a,b] | n.d.[b] |
| 1-Heptanol | 1453 | A | 0.0150 ± 0.0007[a] | 0.0113 ± 0.0007[b] | 0.0145 ± 0.0004[a] | 0.0196 ± 0.0012[c] | 0.0154 ± 0.0019[a] | 0.0143 ± 0.0010[a] |
| 2-Methy-6-hepten-1-ol | 1459 | C | 0.0109 ± 0.0015[a] | 0.0092 ± 0.0024[a] | 0.0122 ± 0.0024[a] | 0.0093 ± 0.0007[a] | 0.0103 ± 0.0017[a] | 0.0108 ± 0.0007[a] |
| 2-Ethyl-1-hexanol | 1486 | A | 0.0334 ± 0.0021[a] | 0.026 ± 0.004[a] | 0.035 ± 0.009[a] | 0.033 ± 0.006[a] | 0.032 ± 0.003[a] | 0.026 ± 0.004[a] |
| 6-Hepten-1-ol | 1509 | C | 0.0294 ± 0.0018[a] | 0.029 ± 0.003[a] | 0.0314 ± 0.0017[a] | 0.0386 ± 0.0015[b] | 0.0323 ± 0.0024[a] | 0.031 ± 0.003[a] |
| 1-Octanol | 1557 | A | 0.0217 ± 0.0009[a] | 0.0185 ± 0.0022[a] | 0.0214 ± 0.0010[a] | 0.025 ± 0.004[a] | 0.025 ± 0.004[a] | 0.0210 ± 0.0021[a] |
| *cis*-5-Octen-1-ol | 1611 | B[4] | 0.0132 ± 0.0006[a,b] | 0.0120 ± 0.0012[a] | 0.0138 ± 0.0005[b] | 0.0156 ± 0.0007[c] | 0.0144 ± 0.0009[b,c] | 0.0134 ± 0.0012[a,b] |
| Furfuryl alcohol | 1660 | A | 0.070 ± 0.023[a] | 0.052 ± 0.008[a] | 0.062 ± 0.005[a] | 0.075 ± 0.015[a] | 0.08 ± 0.05[a] | 0.067 ± 0.011[a] |
| 1-Nonanol | 1663 | A | 0.0090 ± 0.0017[a] | 0.010 ± 0.004[a] | 0.009 ± 0.003[a] | 0.013 ± 0.008[a] | 0.0107 ± 0.0010[a] | 0.0093 ± 0.0016[a] |
| Benzyl alcohol | 1888 | A | 0.113 ± 0.010[a,d] | 0.044 ± 0.004[b] | 0.056 ± 0.011[b] | 0.176 ± 0.016[c] | 0.13 ± 0.03[d] | 0.078 ± 0.008[a,b] |
| 2-Phenylethanol | 1926 | A | 0.73 ± 0.03[a,d] | 0.070 ± 0.008[b] | 0.199 ± 0.014[c] | 0.81 ± 0.07[d] | 0.66 ± 0.04[a,d] | 0.32 ± 0.04[e] |
| 1-Undecanol | 1874 | B[5] | 0.045 ± 0.022[a] | 0.025 ± 0.003[a] | 0.030 ± 0.005[a] | 0.033 ± 0.003[a] | 0.038 ± 0.021[a] | 0.026 ± 0.003[a] |
| 1-Dodecanol | 1978 | B[6] | 0.0033 ± 0.008[a] | 0.014 ± 0.004[a] | 0.033 ± 0.012[a] | 0.039 ± 0.005[a] | 0.03 ±0.03[a] | 0.030 ± 0.012[a] |
| 1-Tetradecanol | 2187 | B[3] | 0.020 ± 0.004[a] | 0.022 ± 0.003[a] | 0.018 ± 0.004[a] | 0.027 ± 0.004[a] | 0.032 ± 0.014[a] | 0.0149 ± 0.0019[a] |
| *Total of alcohols* | | | 4.701[a,b] | 3.115[b] | 4.818[a,b] | 7.879[c] | 5.894[a] | 4,513[a,b] |
| ***Acetic acid esters*** | | | | | | | | |
| Methyl acetate | 815[a] | A | 0.128 ± 0.015[a] | 0.170 ± 0.016[a] | 0.17 ± 0.03[a] | 0.29 ± 0.05[b] | 0.29 ± 0.08[b] | 0.147 ± 0.015[a] |
| Ethyl acetate | 873[a] | A | 0.13 ± 0.03[a] | 0.111 ± 0.004[a] | 0.153 ± 0.007[a] | 1.06 ± 0.12[b] | 0.60 ± 0.11[c] | 0.147 ± 0.022[a] |
| *cis*-3-Hexenyl acetate | 1298 | A | 0.0074 ± 0.0008[a,b] | 0.0059 ± 0.0007[a] | 0.0084 ± 0.0006[b] | 0.0083 ± 0.0006[b] | 0.0081 ± 0.0017[a,b] | 0.0076 ± 0.0009[a,b] |
| 2-Phenylethyl acetate | 1811 | A | 0.0101 ± 0.0009[a] | n.d.[b] | n.d.[b] | 0.0163 ± 0.0008[c] | 0.0105 ± 0.0006[a] | n.d.[b] |
| *Total of acetic acid esters* | | | 0.275[a] | 0.287[a] | 0.328[a] | 1.373[b] | 0.903[c] | 0.302[a] |
| ***Aldehydes*** | | | | | | | | |
| 2-Ethenyl-2-butenal | 1252 | C | 0.0066 ± 0.0005[a] | 0.00665 ± 0.00024[a] | 0.0084 ± 0.0006[a] | n.d.[b] | n.d.[b] | 0.004 ± 0.004[a,b] |
| Octanal | 1270 | A | 0.0055 ± 0.0016[a] | 0.006 ± 0.007[a] | n.d.[a] | 0.008 ± 0.009[a] | n.d.[a] | n.d.[a] |
| Nonanal | 1374 | A | 0.021 ± 0.008[a] | 0.031 ± 0.017[a] | 0.024 ± 0.014[a] | 0.04 ± 0.04[a] | 0.0254 ± 0.0022[a] | 0.030 ± 0.003[a] |
| 2-Furfuraldehyde | 1443 | A | 0.12 ± 0.08[a] | 0.050 ± 0.006[a] | 0.065 ± 0.015[a] | 0.10 ± 0.05[a] | 0.08 ± 0.04[a] | 0.065 ± 0.022[a] |
| Decanal | 1483 | B[7] | 0.013 ± 0.006[a] | 0.019 ± 0.012[a] | 0.021 ± 0.018[a] | 0.03 ± 0.03[a] | 0.018 ± 0.006[a] | 0.0147 ± 0.018[a] |
| Benzaldehyde | 1505 | A | 0.032 ± 0.003[a] | 0.037 ± 0.006[a] | 0.0359 ± 0.0019[a] | 0.041 ± 0.008[a] | 0.041 ± 0.016[a] | 0.034 ± 0.004[a] |
| 5-Methyl-2-furaldehyde | 1561 | A | 0.011 ± 0.004[a] | 0.0070 ± 0.0006[a] | 0.0092 ± 0.0014[a] | 0.010 ± 0.002[a] | 0.011 ± 0.004[a] | 0.0082 ± 0.0011[a] |
| Isoxylaldehyde | 1809 | C | 0.098 ± 0.011[a,b] | 0.10 ± 0.03[a,b] | 0.13 ± 0.04[b] | 0.101 ± 0.025[a,b] | 0.067 ± 0.005[a] | 0.072 ± 0.0015[a] |
| 5-Hydroxymethylfurfural | 2482 | A | 0.029 ± 0.019[a] | 0.0131 ± 0.0008[a] | 0.0148 ± 0.0012[a] | 0.021 ± 0.006[a] | 0.021 ± 0.007[a] | 0.014 ± 0.003[a] |
| *Total of aldehydes* | | | 0.339[a] | 0.269[a] | 0.309[a] | 0.357[a] | 0.265[a] | 0.242[a] |
| ***$C_{13}$-Norisoprenoids*** | | | | | | | | |
| β-Damascenone | 1820 | A | 0.026 ± 0.004[a] | 0.029 ± 0.003[a,b] | 0.030 ± 0.003[a,b] | 0.036 ± 0.004[b] | 0.031 ± 0.003[a,b] | 0.034 ± 0.004[b] |
| 3-Hydroxy-β-damascone | 2505 | C | 0.0547 ± 0.0024[a] | 0.055 ± 0.008[a] | 0.066 ± 0.004[a] | 0.056 ± 0.006[a] | 0.054 ± 0.004[a] | 0.067 ±0.009[a] |
| *Total of $C_{13}$-norisoprenoinds* | | | 0.080[a] | 0.084[a] | 0.096[a,b] | 0.092[a,b] | 0.085[a,b] | 0.101[b] |

Supplementary material to: *Lactic acid bacteria and yeast inocula modulate the volatile profile of Spanish-style green table olive fermentations*

| | | | | | | | | |
|---|---|---|---|---|---|---|---|---|
| ***Ethyl esters*** | | | | | | | | |
| Ethyl lactate | 1333 | A | 0.25 ± 0.08$^{a,b}$ | 0.219 ± 0.015$^{a}$ | 0.28 ± 0.06$^{a,b}$ | 0.35 ± 0.03$^{a,b}$ | 0.38 ± 0.07$^{b}$ | 0.29 ± 0.03$^{a,b}$ |
| Ethyl hydrocinnamate | 1884 | C | 0.009 ± 0.005$^{a}$ | 0.0065 ± 0.0009$^{a}$ | 0.0106 ± 0.0010$^{a}$ | 0.020 ± 0.008$^{a}$ | 0.019 ± 0.005$^{a}$ | 0.010 ± 0.004$^{a}$ |
| Ethyl 5,6-dimethylnicotinate | 2031 | C | 0.0064 ± 0.0015$^{a}$ | 0.0081 ± 0.0022$^{a}$ | 0.015 ± 0.003$^{b}$ | 0.042 ± 0.003$^{c}$ | 0.0098 ± 0.0011$^{a,b}$ | 0.0088 ± 0.0012$^{a,b}$ |
| Unknown ester (m/z 88) | 2253 | - | 0.127 ± 0.017$^{a}$ | 0.154 ± 0.014$^{a,b}$ | 0.1817 ± 0.0013$^{b}$ | 0.125 ± 0.013$^{a}$ | 0.158 ± 0.014$^{b}$ | 0.174 ±0.023$^{b}$ |
| *Total of ethyl esters* | | | 0.393$^{a}$ | 0.387$^{a}$ | 0.491$^{a,b}$ | 0.538$^{a,b}$ | 0.569$^{b}$ | 0.486$^{a,b}$ |
| ***Ketones*** | | | | | | | | |
| Diacetyl | 943$^{a}$ | A | 0.009 ± 0.003$^{a}$ | 0.0066 ± 0.0005$^{a}$ | 0.0080 ± 0.0003$^{a}$ | 0.0098 ± 0.0016$^{a}$ | 0.010 ± 0.003$^{a}$ | 0.0087 ± 0.0016$^{a}$ |
| 4-Methyl-2-pentanone | 981$^{a}$ | C | 0.0285 ± 0.0015$^{a}$ | 0.0282 ± 0.023$^{a}$ | 0.0260 ± 0.0009$^{a}$ | 0.028 ± 0.003$^{a}$ | 0.0290 ± 0.0024$^{a}$ | 0.028 ± 0.003$^{a}$ |
| 2,6-Dimethyl-4-heptanone | 1151 | C | 0.0085 ± 0.0011$^{a}$ | 0.0072 ± 0.0006$^{a}$ | 0.005 ± 0.007$^{a}$ | 0.0099 ± 0.0016$^{a}$ | 0.0095 ± 0.0022$^{a}$ | 0.0096 ± 0.0014$^{a}$ |
| Acetoin | 1277 | A | 0.071 ± 0.003$^{a}$ | 0.08 ± 0.03$^{a}$ | 0.065 ± 0.015$^{a}$ | 0.074 ± 0.010$^{a}$ | 0.142 ± 0.009$^{b}$ | 0.099 ± 0.015$^{a}$ |
| 1-Hydroxy-2-propanone | 1290 | B[8] | 0.46 ± 0.17$^{a}$ | 0.34 ± 0.08$^{a}$ | 0.349 ± 0.018$^{a}$ | 0.41 ± 0.04$^{a}$ | 0.49 ± 0.22$^{a}$ | 0.45 ± 0.08$^{a}$ |
| 6-Methyl-5-hepten-2-one | 1322 | A | 0.0073 ± 0.0008$^{a}$ | n.d.$^{a}$ | 0.0070 ± 0.0019$^{a}$ | 0.005 ± 0.006$^{a}$ | n.d.$^{a}$ | n.d.$^{a}$ |
| 2-Cyclopenten-1-one | 1347 | B[9] | 0.014 ± 0.005$^{a}$ | 0.0090 ± 0.0012$^{a}$ | 0.0112 ± 0.0015$^{a}$ | 0.014 ± 0.004$^{a}$ | 0.012 ± 0.005$^{a}$ | 0.0111 ± 0.0014$^{a}$ |
| 1-Hydroxy-2-butanone | 1362 | B[3] | 0.035 ± 0.020$^{a}$ | 0.020 ± 0.004$^{a}$ | 0.021 ± 0.003$^{a}$ | 0.029 ± 0.006$^{a}$ | 0.030 ± 0.014$^{a}$ | 0.027 ± 0.003$^{a}$ |
| 2-Acetylfuran | 1490 | A | 0.0127 ± 0.0018$^{a}$ | 0.0102 ± 0.0019$^{a}$ | 0.01276 ± 0.00004$^{a}$ | 0.013 ± 0.003$^{a}$ | 0.014 ± 0.006$^{a}$ | 0.0127 ± 0.0018$^{a}$ |
| 6-Methyl-3,5-heptadien-2-one | 1586 | B[3] | 0.0214 ± 0.0004$^{a}$ | 0.0202 ± 0.0016$^{a}$ | 0.026 ± 0.003$^{b}$ | 0.0256 ± 0.0012$^{b}$ | 0.0217 ± 0.0018$^{a}$ | 0.0225 ± 0.0018$^{a,b}$ |
| Acetophenone | 1640 | A | 0.025 ±0.009$^{a}$ | 0.0152 ± 0.0014$^{a}$ | 0.0192 ± 0.0014$^{a}$ | 0.028 ± 0.013$^{a}$ | 0.019 ± 0.007$^{a}$ | 0.0166 ± 0.0022$^{a}$ |
| 1,2-Cyclopentanedione | 1779 | C | 0.025 ± 0.009$^{a}$ | 0.032 ± 0.008$^{a}$ | 0.02 ± 0.03$^{a}$ | 0.026 ± 0.005$^{a}$ | 0.040 ± 0.015$^{a}$ | 0.045 ± 0.020$^{a}$ |
| Cyclotene | 1846 | A | 0.030 ± 0.013$^{a}$ | 0.009 ± 0.003$^{a}$ | 0.0128 ± 0.0021$^{a}$ | 0.014 ± 0.004$^{a}$ | 0.013 ± 0.006$^{a}$ | 0.013 ± 0.003$^{a}$ |
| Purpurocatechol | 2020 | C | 0.0081 ± 0.0009$^{a,c}$ | 0.0070 ± 0.0009$^{a}$ | 0.00933 ± 0.00019$^{a,c}$ | n.d.$^{b}$ | n.d.$^{b}$ | 0.0098 ± 0.0020$^{c}$ |
| *Total of ketones* | | | 0.748$^{a}$ | 0.578$^{a}$ | 0.594$^{a}$ | 0.683$^{a}$ | 0.835$^{a}$ | 0.752$^{a}$ |
| ***Lactones*** | | | | | | | | |
| γ-Butyrolactone | 1624 | A | 0.014 ± 0.006$^{a}$ | 0.0096 ± 0.0016$^{a}$ | 0.0113 ± 0.0011$^{a}$ | 0.012 ± 0.003$^{a}$ | 0.016 ± 0.009$^{a}$ | 0.0111 ± 0.0017$^{a}$ |
| γ-Hexalactone | 1705 | A | 0.0077 ± 0.0007$^{a}$ | 0.0066 ± 0.0005$^{a}$ | 0.0071 ± 0.0011$^{a}$ | n.d.$^{a}$ | 0.004 ± 0.005$^{a}$ | 0.004 ± 0.005$^{a}$ |
| 2(5H)-Furanone | 1754 | A | 0.049 ± 0.013$^{a}$ | 0.039 ± 0.008$^{a}$ | 0.035 ± 0.010$^{a}$ | 0.045 ± 0.005$^{a}$ | 0.06 ± 0.03$^{a}$ | 0.050 ± 0.012$^{a}$ |
| γ-Octalactone | 1930 | B[3] | 0.0106 ± 0.0008$^{a}$ | 0.0094 ± 0.0013$^{a}$ | 0.012 ± 0.004$^{a}$ | 0.0082 ± 0.0012$^{a}$ | 0.011 ± 0.003$^{a}$ | 0.0120 ± 0.0010$^{a}$ |
| δ-Octalactone | 1989 | A | 0.033 ± 0.005$^{a}$ | 0.0265 ± 0.0022$^{a}$ | 0.033 ± 0.005$^{a}$ | 0.0327 ± 0.0011$^{a}$ | 0.029 ± 0.008$^{a}$ | 0.038 ± 0.004$^{a}$ |
| γ-Nonalactone | 2046 | A | 0.024 ± 0.003$^{a}$ | 0.0183 ± 0.0022$^{a}$ | 0.026 ± 0.007$^{a}$ | 0.022 ± 0.003$^{a}$ | 0.027 ± 0.009$^{a}$ | 0.0265 ± 0.019$^{a}$ |
| γ-Decalactone | 2165 | B[3] | 0.0101 ± 0.0018$^{a}$ | 0.003 ± 0.004$^{a}$ | 0.012 ± 0.004$^{a}$ | 0.0087 ± 0.0006$^{a}$ | 0.007 ± 0.008$^{a}$ | 0.0103 ± 0.0019$^{a}$ |
| Iridomyrmecine | 2210 | C | 0.0249 ± 0.0019$^{a,d}$ | 0.0175 ± 0.0025$^{b,c}$ | 0.022 ± 0.003$^{a,c}$ | 0.0281 ± 0.0007$^{d}$ | 0.0150 ± 0.0015$^{b}$ | 0.024 ± 0.003$^{a,d}$ |
| *Total of lactones* | | | 0.175$^{a}$ | 0.131$^{a}$ | 0.158$^{a}$ | 0.157$^{a}$ | 0.171$^{a}$ | 0.176$^{a}$ |
| ***Methyl esters*** | | | | | | | | |
| Methyl lactate | 1309 | B[3] | 0.092 ± 0.008$^{a}$ | 0.113 ± 0.009$^{a}$ | 0.09 ± 0.04$^{a}$ | 0.035 ± 0.006$^{b}$ | 0.094 ± 0.012$^{a}$ | 0.098 ± 0.015$^{a}$ |
| Methyl hydrocinnamate | 1843 | B[3] | 0.012 ± 0.002$^{a,b}$ | 0.0118 ± 0.0009$^{a,b}$ | 0.0143 ± 0.0009$^{b}$ | 0.0085 ± 0.0022$^{a}$ | 0.014 ± 0.003$^{a,b}$ | 0.0131 ± 0.0023$^{b}$ |
| Methyl 4(methylamino)benzoate | 1993 | C | 0.058 ± 0.006$^{a}$ | 0.104 ± 0.025$^{a,b}$ | 0.11 ± 0.05$^{a,b}$ | 0.154 ± 0.008$^{b}$ | 0.069 ± 0.010$^{a}$ | 0.080 ± 0.012$^{a}$ |

| | | | | | | | | |
|---|---|---|---|---|---|---|---|---|
| Methyl 2-formylbenzoate | 2199 | C | 0.017 ± 0.008$^{a}$ | 0.0088 ± 0.0006$^{a}$ | 0.020 ± 0.011$^{a}$ | 0.006 ± 0.007$^{a}$ | 0.004 ± 0.004$^{a}$ | 0.011 ± 0.003$^{a}$ |
| *Total of methyl esters* | | | 0.178$^{a}$ | 0.237$^{a}$ | 0.237$^{a}$ | 0.204$^{a}$ | 0.181$^{a}$ | 0.202$^{a}$ |
| ***Nitrogen compounds*** | | | | | | | | |
| Pyrazyne | 1205 | A | 0.0079 ± 0.0009$^{a}$ | 0.010 ± 0.003$^{a}$ | 0.0072 ± 0.0009$^{a}$ | 0.0078 ± 0.0022$^{a}$ | 0.014 ± 0.008$^{a}$ | 0.008 ± 0.009$^{a}$ |
| 3-Ethylpyridine | 1383 | A | 0.0081 ± 0.0016$^{a,c}$ | 0.009 ± 0.003$^{a,c}$ | 0.0157 ± 0.0020$^{b}$ | 0.014 ± 0.003$^{b,c}$ | 0.0074 ± 0.0007$^{a}$ | 0.012 ± 0.003$^{a,b,c}$ |
| 3-Ethenylpyridine | 1476 | C | 0.0086 ± 0.0021$^{a}$ | 0.0099 ± 0.0024$^{a}$ | 0.01315 ± 0.00012$^{a}$ | 0.0122 ± 0.0022$^{a}$ | 0.0079 ± 0.0007$^{a}$ | 0.010 ± 0.003$^{a}$ |
| Pyrrole | 1495 | B[3] | 0.0103 ± 0.0020$^{a}$ | 0.0096 ± 0.0013$^{a}$ | 0.0097 ± 0.0009$^{a}$ | 0.0108 ± 0.0009$^{a}$ | 0.013 ± 0.007$^{a}$ | 0.010 ± 0.003$^{a}$ |
| *Total of Nitrogen compounds* | | | 0.035$^{a}$ | 0.039$^{a}$ | 0.046$^{a}$ | 0.045$^{a}$ | 0.043$^{a}$ | 0.042$^{a}$ |
| ***Other esters*** | | | | | | | | |
| 2-Ethylhexyl salicylate | 2305 | C | 0.0074 ± 0.0008$^{a}$ | n.d.$^{b}$ | 0.012 ± 0.006$^{a}$ | 0.0086 ± 0.0013$^{a}$ | 0.0109 ± 0.0012$^{a}$ | 0.0110 ± 0.0017$^{a}$ |
| ***Sulfur compounds*** | | | | | | | | |
| Dimethyl sulphide | 815[a] | C | 0.13 ± 0.03$^{a}$ | 0.170 ± 0.022$^{a}$ | 0.139 ± 0.022$^{a}$ | 0.158 ± 0.026$^{a}$ | 0.18 ± 0.04$^{a}$ | 0.14 ± 0.04$^{a}$ |
| Dimethyl Sulfoxide | 1618 | C | 0.0116 ± 0.0023$^{a,b}$ | 0.0186 ± 0.0022$^{b}$ | 0.0110 ± 0.0003$^{a}$ | 0.014 ± 0.004$^{a,b}$ | 0.015 ± 0.005$^{a,b}$ | 0.0148 ± 0.0024$^{a,b}$ |
| *Total of Sulfur compounds* | | | 0.137$^{a}$ | 0.189$^{a}$ | 0.150$^{a}$ | 0.171$^{a}$ | 0.199$^{a}$ | 0.155$^{a}$ |
| ***Terpenes*** | | | | | | | | |
| Dihydromyrcenol | 1466 | B[3] | 0.0087 ± 0.0008$^{a}$ | 0.004 ± 0.005$^{a}$ | 0.0102 ± 0.0005$^{a}$ | 0.006 ± 0.006$^{a}$ | 0.006 ± 0.007$^{a}$ | 0.0089 ± 0.0012$^{a}$ |
| 2-Bornene | 1517 | C | 0.051 ± 0.009$^{a}$ | 0.040 ± 0.004$^{a}$ | 0.042 ± 0.007$^{a}$ | 0.048 ± 0.008$^{a}$ | 0.058 ± 0.015$^{a}$ | 0.047 ± 0.006$^{a}$ |
| Linalool | 1540 | A | 0.0141 ± 0.0008$^{a}$ | 0.0136 ± 0.0019$^{a}$ | 0.07 ± 0.08$^{a}$ | 0.0139 ± 0.0022$^{a}$ | 0.0143 ± 0.0023$^{a}$ | 0.0121 ± 0.0019$^{a}$ |
| α-Isophorone | 1593 | B[3] | 0.0082 ±0.0003$^{a,c}$ | 0.0079 ± 0.0006$^{a}$ | 0.0099 ± 0.0007$^{b}$ | 0.0089 ± 0.0004$^{a,b,c}$ | 0.0081 ± 0.0005$^{a}$ | 0.0092 ± 0.0009$^{b,c}$ |
| α-Terpineol | 1702 | A | 0.0102 ± 0.0010$^{a,b}$ | 0.0078 ± 0.0008$^{a}$ | 0.0079 ± 0.0004$^{a}$ | 0.0083 ± 0.0010$^{a,b}$ | 0.0109 ± 0.0019$^{b}$ | 0.0087 ± 0.0007$^{a,b}$ |
| Geraniol | 1857 | A | 0.0250 ± 0.0012$^{a}$ | 0.0139 ± 0.0014$^{b}$ | 0.01817 ± 0.00013$^{c}$ | 0.0180 ± 0.0018$^{c}$ | 0.023 ± 0.003$^{a}$ | 0.0172 ± 0.0019$^{b,c}$ |
| *Total of terpenes* | | | 0.117$^{a}$ | 0.087$^{a}$ | 0.157$^{a}$ | 0.102$^{a}$ | 0.121$^{a}$ | 0.103$^{a}$ |
| ***Volatile Phenols*** | | | | | | | | |
| Guaiacol | 1863 | A | 0.025 ± 0.008$^{a}$ | 0.016 ± 0.003$^{a}$ | 0.0221 ± 0.0011$^{a}$ | 0.023 ± 0.005$^{a}$ | 0.020 ± 0.004$^{a}$ | 0.023 ± 0.006$^{a}$ |
| 4-Methylguaiacol | 1962 | B[3] | 0.0100 ± 0.0004$^{a}$ | 0.0065 ± 0.0006$^{b}$ | 0.0083 ± 0.0007$^{c}$ | 0.0113 ± 0.0004$^{d}$ | 0.0088 ± 0.0006$^{c}$ | 0.0085 ± 0.0008$^{c}$ |
| 4-Ethylguaiacol | 2038 | A | 0.0354 ± 0.0024$^{a}$ | n.d.$^{b}$ | n.d.$^{b}$ | 0.032 ± 0.006$^{a}$ | 0.018 ± 0.003$^{c}$ | 0.0096 ± 0.0021$^{c}$ |
| 4-Ethylphenol | 2192 | A | 0.102 ± 0.020$^{a,c}$ | 0.13 ± 0.05$^{a,c}$ | 0.51 ± 0.12$^{b}$ | 0.0154 ± 0.0007$^{a}$ | 0.28 ± 0.16$^{b,c}$ | 0.18 ± 0.04$^{a,c}$ |
| Isovanillic acid | 2376 | C | 0.0154 ± 0.0009$^{a,d}$ | 0.0123 ±0.0015$^{a,c}$ | 0.0191 ± 0.0007$^{b}$ | 0.0204 ± 0.0025$^{b}$ | 0.0104 ± 0.0008$^{c}$ | 0.0178 ± 0.0017$^{b,d}$ |
| Coumaran | 2409 | C | 0.034 ± 0.004$^{a}$ | 0.041 ± 0.005$^{a,b}$ | 0.046 ± 0.007$^{b}$ | 0.047 ± 0.003$^{b}$ | 0.041 ± 0.005$^{a,b}$ | 0.077 ± 0.011$^{c}$ |
| 5-*tert*-Butylpyrogallol | 2419 | C | 0.0099 ± 0.0008$^{a,d}$ | n.d.$^{b}$ | n.d.$^{b}$ | 0.0133 ± 0.0012$^{a}$ | 0.003 ± 0.004$^{b,c}$ | 0.0082 ± 0.0014$^{c,d}$ |
| Methoxyeugenol | 2497 | C | 0.031 ± 0.003$^{a,b}$ | 0.026 ± 0.004$^{a,d}$ | 0.041 ± 0.006$^{b,c}$ | 0.049 ± 0.007$^{c}$ | 0.0179 ± 0.0020$^{d}$ | 0.038 ± 0.004$^{b,c}$ |
| Vainillin | 2516 | A | 0.0366 ± 0.0017$^{a,b}$ | 0.035 ± 0.005$^{a,b}$ | 0.023 ± 0.013$^{a}$ | 0.025 ± 0.003$^{a}$ | 0.030 ± 0.003$^{a,b}$ | 0.040 ± 0.005$^{b}$ |
| *Total of volatile phenols* | | | 0.299$^{a}$ | 0.270$^{a}$ | 0.674$^{b}$ | 0.237$^{a}$ | 0.430$^{a}$ | 0.399$^{a}$ |
| ***Other compounds*** | | | | | | | | |
| Furfuryl methyl ether | 1744 | C | 0.0085 ± 0.0004$^{a}$ | 0.0068 ± 0.0011$^{a}$ | 0.0106 ± 0.0014$^{a}$ | 0.0093 ± 0.0018$^{a}$ | n.d.$^{b}$ | 0.0103 ± 0.0025$^{a}$ |
| ***Unknown compounds*** | | | | | | | | |

| | | | | | | | | |
|---|---|---|---|---|---|---|---|---|
| Unknown A (m/z 71-59) | 1026 | - | 0.0106 ± 0.0010[a] | 0.01136 ± 0.00018[a] | 0.0113 ± 0.0008[a] | 0.0084 ± 0.0009[b] | 0.0112 ± 0.0015[a] | 0.01049 ± 0.00009[a] |
| Unknown B (m/z 123-138-96) | 1304 | - | 0.0073 ± 0.0010[a] | 0.0060 ± 0.004[a] | 0.0107 ± 0.0015[a,b] | 0.015 ± 0.004[b] | 0.0107 ± 0.0011[a,b] | 0.0096 ± 0.0018[a,b] |
| Unknown C (m/z 83-112-97) | 1710 | - | 0.002 ± 0.003[a] | n.d.[a] | n.d.[a] | 0.0083 ± 0.0006[b] | n.d.[a] | n.d.[a] |
| Unknown D (m/z 55-93-108) | 1783 | - | 0.172 ± 0.024[a] | 0.078 ± 0.006[b] | 0.112 ± 0.020[b] | 0.59 ± 0.03[c] | 0.35 ± 0.04[d] | 0.099 ± 0.013[b] |
| Unknown E (m/z 111-198) | 1867 | - | 0.0102 ±0.0014[a] | n.d.[a] | 0.012 ± 0.003[a] | 0.031 ± 0.011[b] | 0.0114 ± 0.0011[a] | 0.0090 ± 0.0010[a] |
| Unknown F (m/z 95-154-110) | 2081 | - | 0.008 ± 0.003[a] | n.d.[a] | n.d.[a] | 0.029 ± 0.007[b] | 0.020 ± 0.003[b] | n.d.[a] |
| Unknown G (m/z 138) | 2132 | - | 0.0119 ± 0.0009[a,c] | 0.0106 ± 0.0011[a] | 0.015 ± 0.003[b,c] | 0.0155 ± 0.0005[b] | 0.0103 ± 0.0008[a] | 0.0149 ± 0.0020[b,c] |
| Unknown H (m/z 113-81-153) | 2146 | - | 0.0173 ± 0.0014[a] | 0.0100 ± 0.0008[b] | 0.0137 ± 0.0015[c] | 0.0171 ± 0.0007[a,d] | 0.0146 ± 0.0015[c,d] | 0.0134 ± 0.0017[c] |
| Unknown I (m/z 99-139-67-81) | 2158 | - | 0.070 ± 0.005[a,b] | 0.058 ± 0.006[a,c] | 0.0709 ± 0.0012[b] | 0.049 ± 0.005[c] | 0.065 ± 0.006[a,b] | 0.068 ± 0.009[a,b] |
| Unknown J (m/z 179-148-120) | 2158 | - | 0.0138 ± 0.0020[a] | 0.038 ± 0.005[a] | 0.047 ± 0.021[a] | 0.06 ± 0.06[a] | 0.0257 ± 0.0020[a] | 0.039 ± 0.009[a] |
| Unknown K (m/z 93-79) | 2195 | - | 0.0190 ± 0.0011[a,c] | 0.0132 ± 0.0021[b,d] | 0.021 ± 0.003[c] | 0.0161 ± 0.0010[a,d] | 0.0098 ± 0.0010[b] | 0.0173 ± 0.0024[a,c,d] |
| Unknown L (m/z 222-43-85-177) | 2200 | - | 0.025 ± 0.003[a] | 0.028 ± 0.003[a,b] | 0.030 ± 0.004[a,b] | 0.0283 ± 0.0020[a,b] | 0.0256 ± 0.0021[a] | 0.036 ± 0.008[b] |
| Unknown M (m/z 138-120) | 2216 | - | 0.0290 ± 0.0020[a,c] | 0.0266 ± 0.0024[a] | 0.036 ± 0.003[b,c] | 0.035 ± 0.004[a,b,c] | 0.028 ± 0.004[a,c] | 0.038 ± 0.005[b] |
| Unknown N (m/z 151-43) | 2227 | - | 0.0214 ± 0.0016[a] | 0.0215 ± 0.0022[a] | 0.0213 ± 0.0005[a] | 0.0110 ± 0.0017[b] | 0.0201 ± 0.0025[a] | 0.022 ± 0.004[a] |
| Unknown O (m/z 95-110-138) | 2236 | - | 0.0106 ± 0.0010[a] | 0.0099 ± 0.0009[a] | 0.0124 ± 0.0018[a,b] | 0.0105 ± 0.0012[a] | 0.0104 ± 0.0009[a] | 0.0138 ± 0.0018[b] |
| Unknown P (m/z 138) | 2255 | - | 0.0166 ± 0.012[a] | 0.0168 ± 0.0018[a] | 0.023 ± 0.003[b] | 0.0215 ± 0.0017[b] | 0.0164 ± 0.0016[a] | 0.022 ± 0.003[b] |
| Unknown Q (m/z 102-55-69) | 2281 | - | 0.030 ± 0.012[a] | 0.031 ± 0.003[a,e] | 0.056 ± 0.007[b,d] | 0.134 ± 0.006[c] | 0.073 ± 0.009[d] | 0.050 ± 0.008[b,e] |
| Unknown R (m/z 85-128) | 2284 | - | 0.0087 ± 0.0024[a] | n.d.[a] | 0.0081 ± 0.0007[a] | 0.0082 ± 0.0006[a] | 0.006 ± 0.007[a] | 0.0092 ± 0.0012[a] |
| Unknown S (m/z 167-121) | 2431 | - | 0.0285 ± 0.0025[a] | 0.0183 ± 0.0017[b] | 0.0230 ± 0.0017[c,d] | 0.0267 ± 0.0018[a,c,d] | 0.027 ± 0.003[a,d] | 0.025 ± 0.003[c,d] |
| Unknown T (m/z 70-55-82) | 2467 | - | 0.252 ± 0.006[a] | 0.18 ± 0.03[a,b] | 0.1758 ± 0.0018[a,b] | 0.16 ± 0.03[b] | 0.197 ± 0.017[a,b] | 0.23 ± 0.07[a,b] |
| Unknown U (m/z 119-159-192) | 2518 | - | 0.0464 ± 0.0025[a,b] | 0.049 ± 0.005[a,b] | 0.058 ± 0.008[b] | 0.036 ± 0.006[a] | 0.045 ± 0.006[a,b] | 0.057 ± 0.007[b] |
| Unknown V (m/z 189-204) | 2537 | - | 0.029 ± 0.003[a] | 0.025 ± 0.003[a] | 0.035 ± 0.012[a] | 0.0334 ± 0.0013[a] | 0.027 ± 0.003[a] | 0.030 ± 0.003[a] |
| Unknown W (m/z 121-136-161) | 2581 | - | 0.122 ± 0.009[a] | 0.088 ± 0.011[b] | 0.109 ± 0.004[a,b] | 0.124 ± 0.015[a] | 0.115 ± 0.011[a] | 0.119 ± 0.012[a] |
| ***Total of volatile compounds*** | | | 8.82[a,b] | 6.67[b] | 9.18[a,b] | 13.49[c] | 11.19[a,c] | 8.73[a,b] |

[a] LRI values estimated by linear regression.

ID: reliability of identification: A, mass spectrum and LRI agreed with standards; B, mass spectrum agreed with mass spectral data base and LRI agreed with the literature data; C, mass spectrum agreed with mass spectral data base.

[b]Literature reference agreed with LRI data: 1) Ledauphin et al., 2004; 2) Siegmund, Derler, & Pfannhauser, 2001; 3) National Center for Biotechnology Information , 2005; 4) Karimi & Ito, 2012; 5) Tabanca, Demirci, Crockett, Baser, & Wedge, 2007; 6) Shimoda, Nakada, Nakashima, & Osajima, 1997; 7) Nielsen & Poll, 2004; 8) Pozo-Bayon, Ruiz-Rodriguez, Pernin, & Cayot, 2007; 9) Werkhoff , Guntert, Krammer, Sommer, & Kaulen, 1998.

[c]Similar superscript letter in the same row indicates no significant statistically differences (p<0,05).

n.d.: peak not detected.

Supplementary material to: *Lactic acid bacteria and yeast inocula modulate the volatile profile of Spanish-style green table olive fermentations*

References of Table S1:


Karimi, A.G., & Ito, M. (2012). Sedative effect of vapor inhalation of essential oil from Heracleum afghanicum Kitamura seeds. *Journal of Essential Oil Research, 24*, 571–577. https://doi.org/10.1080/10412905.2012.728085.

Ledauphin, J., Saint-Clair, J. F., Lablanquie, O., Guichard, H., Founier, N., Guichard, E., & Barillier, D. (2004). Identification of Trace Volatile Compounds in Freshly Distilled Calvados and Cognac Using Preparative Separations Coupled with Gas Chromatography-Mass Spectrometry. *Journal of Agriculture and Food Chemistry, 52*, 5124–5134. https://doi.org/10.1021/jf040052y.

National Center for Biotechnology Information (2005). PubChem Database. https://pubchem.ncbi.nlm.nih.gov (accessed on June 11, 2019).

Nielsen, G. S., & Poll, L. (2004). Determination of odor active aroma compounds in freshly cut leek (*Allium ampeloprasum* Var. Bulga) and in long-term stored frozen unblanched and blanched leek slices by gas chromatography olfactometry analysis. *Journal of Agriculture and Food Chemistry, 52*, 1642–1646. https://doi.org/10.1021/jf030682k.

Pozo-Bayon, M. A., Ruiz-Rodriguez, A., Pernin, K., & Cayot, N. (2007). Influence of eggs on the aroma composition of a sponge cake and on the aroma release in model studies on flavored sponge cakes. *Journal of Agriculture and Food Chemistry*, *55*, 1418–1426. https://doi.org/10.1021/jf062203y.

Shimoda, M., Nakada, Y., Nakashima, M., & Osajima, Y. (1997). Quantitative comparison of volatile flavor compounds in deep-roasted and light-roasted sesame seed oil. *Journal of Agriculture and Food Chemistry*, *45*, 1997, 3193–3196. https://doi.org/10.1021/jf970172o.

Supplementary material to: *Lactic acid bacteria and yeast inocula modulate the volatile profile of Spanish-style green table olive fermentations*


Siegmund, B., Derler, K., & Pfannhauser, W. (2001). Changes in the aroma of a strawberry drink during storage. *Journal of Agriculture and Food Chemistry, 49*, 2001, 3244–3252. https://doi.org/10.1021/jf010116u.

Tabanca, N., Demirci, B., Crockett, S. L., Baser, K. H. C., & Wedge, D. E. (2007). Chemical composition and antifungal activity of Arnica longifolia, Aster hesperius, and Chrysothamnus nauseosus essential oils. *Journal of Agriculture and Food Chemistry, 55*, 8430–8435. https://doi.org/10.1021/jf010116u.

Werkhoff, P., Guntert, M., Krammer, G., Sommer, H., & Kaulen, J. (1998). Vacuum headspace method in aroma research: flavor chemistry of yellow passion fruits. *Journal of Agriculture and Food Chemistry, 46*, 1076–1093. https://doi.org/10.1021/jf970655s.
